# Children’s Reasoning About Empathy and Social Relationships

**DOI:** 10.1162/opmi_a_00109

**Published:** 2023-10-27

**Authors:** Alexis S. Smith-Flores, Gabriel J. Bonamy, Lindsey J. Powell

**Affiliations:** Department of Psychology, University of California, San Diego

**Keywords:** empathy, relationships, joint reasoning

## Abstract

Across the lifespan, empathic and counter-empathic emotions are shaped by social relationships. Here we test the hypothesis that this connection is encoded in children’s intuitive theory of psychology, allowing them to predict when others will feel empathy versus counter-empathy and to use vicarious emotion information to infer relationships. We asked 4- to 7-year-old children (N = 79) to make emotion predictions or relationship inferences in response to stories featuring two characters, an experiencer and an observer, and either a positive or negative outcome for the experiencer. In the context of positive outcomes, we found that children engaged in robust joint reasoning about relationships and vicarious emotions. When given information about the characters’ relationship, children predicted empathy from a friendly observer and counter-empathy from a rival observer. When given information about the observer’s response to the experiencer, children inferred positive and negative relationships from empathic and counter-empathic responses, respectively. In the context of negative outcomes, children predicted that both friendly and rival observers would feel empathy toward the experiencer, but they still used information about empathic versus counter-empathic responses to infer relationship status. Our results suggest that young children in the US have a blanket expectation of empathic concern in response to negative outcomes, but otherwise expect and infer that vicarious emotions are connected to social relationships. Future research should investigate if children use this understanding to select social partners, evaluate their own relationships, or decide when to express empathy toward others.

## INTRODUCTION

Think about the last few times you felt happy. Likely, some of those instances were the result of a friend or family member’s good news. People’s emotions result not only directly from their own personal experiences, but also from witnessing the experiences of others. Emotions felt as a result of someone else’s experiences are known as “vicarious emotions” (Wondra & Ellsworth, 2015). Vicarious emotions can vary both in valence and in their congruence with the likely emotions of the person who inspires them. We will use the term “empathy” to refer to congruent positive or negative emotions for those having good or bad experiences, respectively (Batson et al., 2009). In contrast, counter-empathy involves feeling incongruent emotions toward others’ experiences, such as sadness in response to others’ good outcomes (i.e., gluckschmerz) or happiness in response to others’ bad outcomes (i.e., schadenfreude) (Smith & van Dijk, 2018).

Empathy and counter-empathy tend to be applied selectively: People often feel more empathy toward those they care more about and feel counter-empathy toward disliked individuals or outgroups (Batson, 2009; Cikara & Fiske, 2012; Masten et al., 2010; Shamay-Tsoory et al., 2014). As a result, expressions of empathy and counter-empathy may be predicted by, and carry information about, a person’s social relationships. Understanding these connections could allow people to effectively seek social support, and to evaluate their own and others’ relationships based on others’ vicarious emotional expressions. Relatively little research has investigated how people think about the role of relationships in vicarious emotions, however, or how such reasoning develops. Here we consider how common aspects of intuitive psychology, available in early childhood, may allow children to engage in systematic reasoning about the connections between vicarious emotions and social relationships. We then test their ability to engage in this kind of reasoning.

Reasoning about others’ emotions often involves considering their desires. Emotions arise from appraisals of the world relative to what the appraiser wants, needs, and expects. When goals are met and desires are fulfilled, observers expect positive responses, while failures and undesirable outcomes are often accompanied by negative responses (Moors, 2013; Oatley & Johnson-Laird, 2014). This dependence on individuals’ goals and preferences results in appraisals that are person-specific: One person’s good outcome may be someone else’s bad outcome, resulting in differing appraisals of the same situation (Manstead & Fischer, 2001; Siemer et al., 2007). For example, the outcome of a political election often excites some people and disappoints others, depending on preferences for different candidates or policies. An observer’s successful emotion prediction thus requires integrating the observed state of the world and the specific desires of the target person. An understanding of the appraisal process also allows for inverse reasoning about the causes of emotions. Based on an emotional expression, an observer who knows the emoter’s desires can infer the experience that led to the expression, while an observer who knows what experience led to the expression could infer the emoter’s desires (Ong et al., 2015; Wu & Schulz, 2018). Even young children are able to use emotion vocalizations to infer unobserved causes (Smith-Flores & Feigenson, 2022; Wellman et al., 2000; Wu et al., 2017), suggesting that the ability to reason about emotion as an appraisal process appears early in development.

Understanding emotions as the outcome of an appraisal process may also support reasoning about vicarious emotions and their connection to social relationships (Smith-Flores & Powell, 2023). Concepts of social relationships involve the expectation that relationship partners care about one another’s goals and welfare (Afshordi & Liberman, 2021; Jern & Kemp, 2014; Powell, 2022). Friends and group members are generally expected to do more to help one another, and both adult and child observers infer relationships from the effort or partiality of offered support (Earp et al., 2021; Liberman & Shaw, 2017; Marshall et al., 2022; Olson & Spelke, 2008). Even infants expect affiliates to support each other when in need (Jin & Baillargeon, 2017; Pun et al., 2021). Conversely, concepts of negative relationships entail expectations of conflict and spite, reflecting a desire for the other person to suffer or fail (Pietraszewski, 2021). Observers may expect these vicarious desires to serve as the basis for appraisals that lead to vicarious emotions. In addition to predicting vicarious emotions, an observer operating with this intuitive theory should also be able to infer what type of relationship led to an expressed vicarious emotion. Upon witnessing a counter-empathic response, indicating opposite appraisals for the experiencer and the emoter, an observer should infer a negative relationship between the two individuals.

There is some evidence that both adults and children reason about the connection between vicarious emotions and social motivations, though most work focuses more on inferences of general social traits as opposed to specific social relationships. Heyman and Gelman (1998) found that children used a person’s pro- or antisocial motives to infer how pleased they were by positive or negative outcomes for the targets of those motives. Wang and Todd (2021) found that adults evaluate a person who empathizes with a valued target person (e.g., a children’s hospital worker) as being warmer, more likable, and more respectable than a non-empathizer. Three- to five-year-old children similarly find empathy for others’ suffering to be normative, which indicates that young children recognize and reason about others’ empathic concern (Paulus et al., 2020). When directed toward morally repugnant or disliked targets, however, adults may prefer those who are indifferent rather than empathic (Krems et al., 2023; Wang & Todd, 2021). This could reflect a dispreference for those who positively appraise outcomes one considers to be either morally wrong or disadvantageous, as well as a desire for partiality in friendships. In other work, adults use emotional expressions (e.g. of anger, sadness, or happiness), either alone or in social contexts, to infer emoters’ aggression, warmth, and affiliative tendencies (Hareli & Hess, 2010; Knutson, 1996). Similar findings extend to people’s evaluations of their own real-world social partners: physicians who empathize with their patients are viewed as more trustworthy (Kim et al., 2004), and perceived empathy between romantic partners is positively related to relationship satisfaction (Cramer, 2003; Cramer & Jowett, 2010; Gable et al., 2004), though it is unclear whether preferences for empathy in these social relationships reflect dispositional or relationship-based reasoning. Together, these findings suggest that adults and children make dispositional judgments about others using observations of their vicarious emotions. They fall short, however, of providing evidence for a systematic framework connecting vicarious emotions to relationship-based appraisals of outcomes.

One set of looking time experiments tested 10- to 11-month-old infants’ expectations about vicarious emotions across different relationship contexts (Smith-Flores et al., 2023). Infants were introduced to a pair of characters with either a positive or a negative relationship, followed by trials in which one character attempted a goal-directed action and the other character responded happily or sadly to the outcome. In the context of a positive relationship and successful outcome, infants looked significantly less at happy than sad response trials, consistent with an expectation for the observer to emote happily to their friend’s success. In the context of a negative relationship, infants’ looking was not different across happy and sad response trials, suggesting no expectation of positive empathy for a rival. The interaction between relationship context and trial type was significant, supporting the conclusion that infants around one year of age do take relationships into account in their expectations of vicarious emotions. However, there was no evidence that infants expected negative relationships to lead to counter-empathic emotions. This may reflect limited early reasoning about vicarious emotions, focused on predicting empathic responses. Alternatively, infants may struggle more generally with predicting negative emotions in such contexts; they also fail to expect that a friendly observer or even the actor will respond sadly to a failed attempt (Skerry & Spelke, 2014; Smith-Flores et al., 2023). By age 4, children are able to make robust, appraisal-based predictions about both positive and negative emotions (Fabes et al., 1991; Wellman et al., 2000), and appear to possess an emerging norm of empathic concern (Paulus et al., 2020). Four-year-old children are also able to reason about representations of emotions that are at odds with the current state of the world (Smith-Flores & Feigenson, 2021). Investigating children’s verbal predictions about vicarious emotions beginning at this age will thus allow us to explore reasoning about a more comprehensive combination of relationships, outcome valence, and vicarious responses. We also test children’s inferences about relationships from information about vicarious emotional responses.

The current study investigates how 4- to 7-year-old children reason about relationships and expressions of empathy. Children were told four stories in which they were asked to predict how an observer would feel about an outcome for another person (i.e., their friend or rival; Empathy Block), and another four stories in which they were asked to infer the relationship between two characters after being given information about the observer’s empathic or counter-empathic response to the other person’s outcome (Affiliation Block). We preregistered three primary hypotheses on the Open-Science Framework (OSF; https://osf.io/d57ny).

First, we hypothesized that children would predict that observers will respond empathically to their positive affiliates (i.e., friends). If this is true, then when children hear stories featuring two friends, they should be more likely to predict that the observing individual will respond to their friend’s outcome with a congruent emotion (i.e., happiness following a good outcome or sadness following a bad outcome) than an incongruent emotion. However, it is possible that children may expect that friends should respond with positive emotion toward their positive affiliates regardless of the outcome. If this is true, then children may predict that the observer will respond with a positive emotion regardless of the valence of their friend’s experience.

Second, we hypothesized that children’s predictions of empathy will be weaker for rivals. If this is true, then children should predict less outcome-congruent emotion when making predictions in stories about rivals as compared to friends, regardless of the outcome type. An alternative hypothesis not anticipated in our preregistration is that children’s possession of strong norms about responding empathically toward others’ suffering may lead them to predict similar levels of empathy following negative outcomes, regardless of the characters’ relationship (Paulus et al., 2020). Young children tend to use norms rather than other factors, including preferences, to predict others’ behavior, and may even say that impermissible actions are impossible (Kalish & Shiverick, 2004; Shtulman & Phillips, 2018; Smith et al., 2009).

Finally, we hypothesized that children will use vicarious emotions to infer social relationships. If so, children should be more likely to infer that two characters have a close positive relationship after hearing that one character responded empathically to the other character. In contrast, children should select responses that correspond to more distant, negative relationships after hearing about a character that responded counter-empathically to another character. Of course, it is also possible that children may use the valence of the emotion response to make inferences about others’ relationships. If this is true, then children may infer that when a character responds negatively to another character, regardless of the outcome of the event, that the two are negatively affiliated, while characters who respond positively are positively affiliated.

We also conducted exploratory analyses on age-related changes in reasoning about vicarious emotions and relationships. Although we had no a priori hypotheses about developmental change, several patterns were possible. First, there may be no change with age, either because children at all ages are at chance or because children make mature systematic predictions from age 4. Next, there could be development both in terms of children’s differential prediction of vicarious emotion depending on relationships and in their inference of relationship valence from vicarious responses. This would indicate that younger children have a weaker understanding of how vicarious emotions are shaped by relationships, reflected in both their predictions of such emotions and inferences from them. Finally, we could observe developmental change in children’s predictions of vicarious emotions but not their inferences from them. This hypothesis is suggested by past results showing that young children often predict that people will behave according to rules and norms, and sometimes even say that it is impossible to do otherwise (DeJesus et al., 2014; Kalish & Shiverick, 2004; Shtulman & Phillips, 2018; Smith et al., 2013). This tendency to predict strong adherence to norms diminishes with age. Because children view empathy for others’ pain as normative (Paulus et al., 2020), younger children may be less likely to predict counter-empathy, despite being able to reason about the meaning of counter-empathic responses for underlying social relationships.

## METHOD

### Participants

Seventy-nine children, aged 4- to 7-years-old (*M*_age_ = 5.93 years; range = 3.98 years to 7.88 years; 42 girls), participated at a science museum in Southern California. One additional child participated but was excluded for being outside the preregistered age range.

Caregivers completed an optional demographic form at the time of participation. Fifty-one children were identified by their caregivers as White, seven as Asian, 1 as American Indian/Alaskan Native, 18 were identified as belonging to two or more races, and two caregivers declined to answer. Fifteen children were identified as Hispanic/Latinx and five caregivers declined to answer. Seventy-one children came from families where at least one caregiver had a college degree or higher, five had at least one caregiver who completed some college, one had at least one caregiver who had a high school diploma or GED, and two caregivers declined to answer.

Our data collection plan was preregistered as follows: We ran data collection sessions at a local science museum until we had at least 72 participants with valid data for both blocks, while also planning to include data from participants with only one valid block and from participants collected in the final testing session required to reach the target sample size. This resulted in unequal numbers of participants included in the two blocks. Seventy-seven participants completed the Empathy Block and 74 participants completed the Affiliation Block.

Participants received a small gift (i.e., temporary tattoos or stickers) to thank them for their participation.

### Materials & Procedures

All materials including preregistrations, data, and analysis code are available on OSF (https://osf.io/d57ny). Participants were read 4 stories per block across 2 blocks, for a total of 8 stories. Each of the four stories featured two characters, an actor and an observer. Stories in the Empathy Block presented participants with information about the relationship between two characters and about a positive or negative outcome for one of the two characters (the actor), before asking participants to predict the emotion felt by the other character (the observer) following that outcome. Stories in the Affiliation Block presented participants with information about a positive or negative outcome for one character (the actor) and the emotion felt by the other character (the observer) following that outcome, before asking participants to infer the degree of affiliation (i.e., the closeness of the relationship) between the two characters. Each block also included an initial warm-up trial that familiarized participants with the relevant response scale (a positive to negative affect scale in the Empathy Block; a positive to negative relationship scale in the Affiliation Block) and tested their ability to use it in responding to questions posed by the experimenter. Participants were randomly assigned to 1 of 8 story orders that counterbalanced block order (Empathy Block first vs. second), outcome order (positive outcome first vs. second), and which affiliation or emotion type came first in each block (friendship/empathy vs. rivalry/counter-empathy).

### Empathy Block

**Figure 1.**
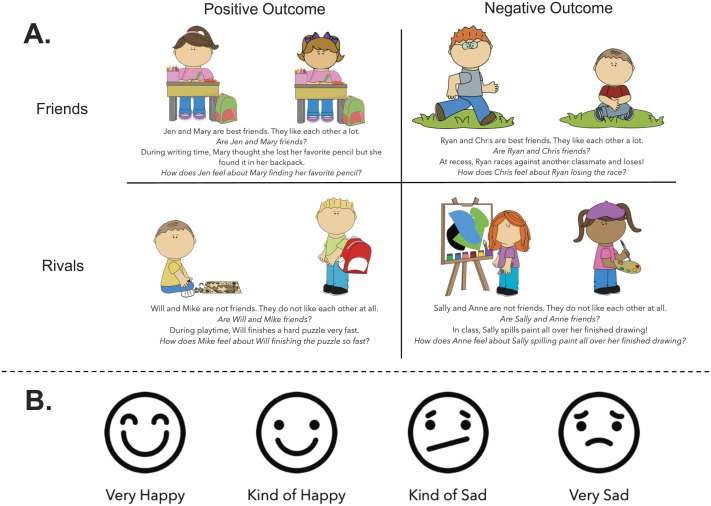
**Stimuli from the Empathy Block.** (A) Children heard 4 stories in total where the actor’s outcome (positive, negative) and characters’ affiliation (friends, rivals) varied across stories. (B) Children were asked to predict how the observer felt about the actor’s outcome using a 4-point scale ranging from “very happy” to “very sad”.

The researcher began by introducing a 4-point Likert scale, ranging from “very happy” to “very sad”, accompanied by simple, line-drawn faces depicting each emotion (Figure 1B). The researcher asked participants about their favorite and least favorite snack. Participants then rated, verbally or by pointing to the scale, how they would feel if they received a lot of their favorite or least favorite snack. As preregistered, participants who responded with the same emotion regarding their favorite and least favorite snack were excluded from this block’s analysis (see Supplemental Information for number of children excluded from each block, Table S1).

Participants were first introduced to the two characters by name and were told about their relationship (e.g., “Ryan and Chris are best friends. They like each other a lot.”). To ensure participants remembered the relationship between the two characters, the researcher then asked a manipulation check question (e.g., “Remind me, are Ryan and Chris friends?”). Participants who did not answer the manipulation check correctly were told the story again and had the manipulation check repeated (Table S2). Participants who did not answer the manipulation check correctly a second time were excluded from that question’s analysis (see Supplemental Information for number of children who failed the manipulation check once or twice for each story, Table S2).

Following the manipulation check question, participants were then told about an event that happened to the actor while the observer watched (e.g., “At recess, Ryan races against another classmate and loses!”). The researcher then asked the test question regarding how the observer felt about the actor’s outcome (e.g., “How does Chris feel about Ryan losing the race?”). The experimenter pointed to each item on the emotion scale, reading the options aloud to the participant. Participants’ verbal or pointed answers were recorded by a second researcher who observed from across the table. A 2 × 2 design was used so that each child heard one story each about a positive outcome for a friend, a negative outcome for a friend, a positive outcome for a rival, and a negative outcome for a rival.

### Affiliation Block

**Figure 2.**
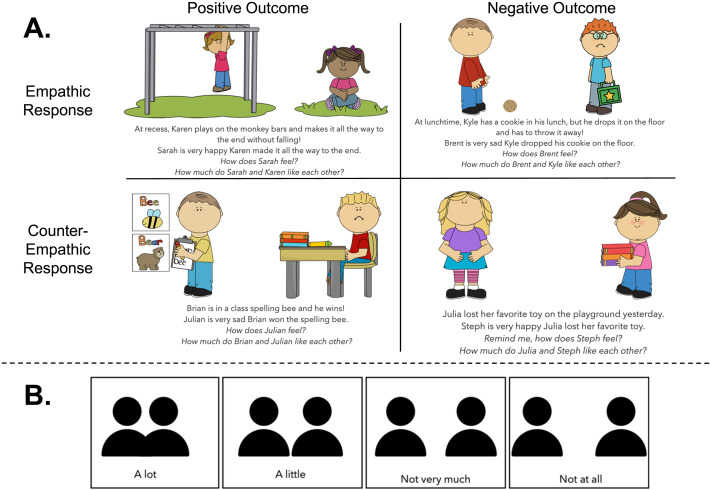
**Stimuli from the Affiliation Block.** (A) Children heard 4 stories in total where the actor’s outcome (positive, negative) and the observer’s emotion (empathic, counter-empathic) changed across stories. (B) Children were asked to infer how much the observer and the actor liked each other using a 4-point scale ranging from “a lot” to “not at all”.

The researcher started by introducing a 4-point Likert scale, used to ask how much people like one another and ranging from “a lot” to “not at all”, accompanied by pictures of two people close together or increasingly far apart (Figure 2B). To familiarize children with this affiliation scale, we asked participants about their favorite and least favorite characters. Participants then rated, verbally or by pointing to the scale, how much they like their favorite or least favorite character. As preregistered, participants who answered the same regarding their favorite and least favorite characters were excluded from this block’s analysis (see Supplemental Information for number of children excluded from each block, Table S1).

Participants were first introduced to the two characters by name and then were told about an event that happened to the actor while the observer watched (e.g., “At lunchtime, Kyle has a cookie in his lunch, but he drops it on the floor and has to throw it away!”; see Figure 2A). Participants were then told how the observer felt about what had happened to the actor (e.g., “Brent is very sad Kyle can’t eat his cookie”). The observer either felt very happy or very sad about the event that happened to the actor.

To ensure participants remembered the emotion felt by the observer, the researcher then asked a manipulation check question (e.g., “Remind me, how does Brent feel?”). Participants who did not answer the manipulation check correctly were told the story again and had the manipulation check repeated (Table S2). Participants who did not answer the manipulation check correctly a second time were excluded from that question’s analysis (see Supplemental Information for number of children who failed the manipulation check once or twice for each story, Table S2).

Finally, the researcher asked the test question regarding the affiliation between the two characters (e.g., “How much do Brent and Kyle like each other?”). The experimenter pointed to each item on the affiliation scale, reading the options aloud to the participant. Participants’ verbal or pointed answers were recorded by a second researcher who observed from across the table. A 2 × 2 design was used so that each participant heard one story each about a positive outcome for the actor with a positive reaction from the observer, a negative outcome with a negative reaction, a positive outcome with a negative reaction, and a negative outcome with a positive reaction.

#### Coding.

All responses were coded by the second researcher at the time of the experiment. An additional coder coded 20% of participants from video recordings to ensure reliability. Agreement between coders was 100%.

For analysis of data from the Empathy Block, participants’ raw emotion predictions were transformed to reflect the degree of expected empathy in the emotion prediction. Responses that indicated the greatest amount of predicted empathy (i.e., “very sad” to a negative outcome and “very happy” to a positive outcome) were coded as a 4, while responses that indicated the greatest predicted counter-empathy (i.e., “very happy” to a negative outcome and “very sad” to a positive outcome) were coded as a 1. Moderately empathic or counter-empathic responses were coded as a 3 or 2, respectively.

In the Affiliation Block, responses that indicated the strongest positive relationship inferences (i.e., the characters like one another “a lot”) were coded as a 4, while responses that indicated the strongest negative relationship inferences (i.e., the characters like one another “not at all”) received a 1. Moderately positive or negative responses were coded as a 3 or 2, respectively.

## RESULTS

### Empathy Block

**Figure 3.**
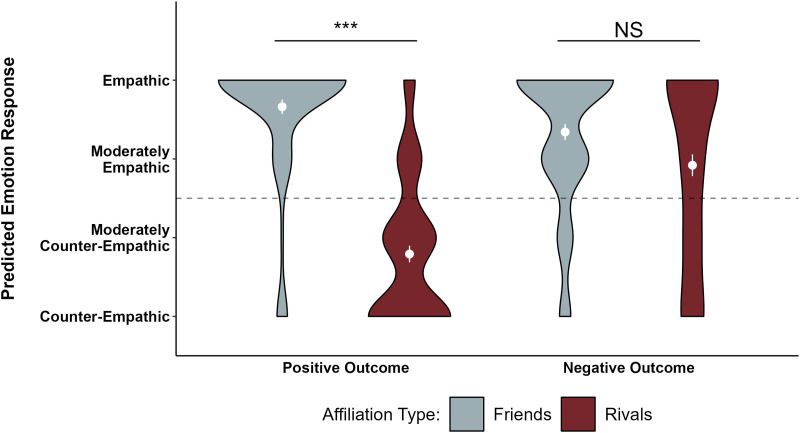
**Children’s responses for each question in the Empathy Block.** The means and standard errors are plotted in white. The dashed line represents the mid-point of the scale (2.5). Significance asterisks come from post-hoc t-tests with bonferroni adjustment as a result of a significant interaction between affiliation type and outcome type. ***p < .001.

First, to test our preregistered hypothesis that children predict that friends will respond empathically toward each other’s outcomes, we conducted separate, two-tailed one-sample t-tests from chance (2.5 on a 1–4 point scale) on children’s responses in the Empathy Block for each of the two stories that featured “best friends”. Regardless of the type of outcome (i.e., positive or negative), children predicted that the observing friend would respond empathically (positive outcome: *M* = 3.662, *SEM* = 0.092, *t*(76) = 12.674, *p* < .001, 95% CI [3.480, 3.845], *d* = 1.444.; negative outcome: *M* = 3.342, *SEM* = 0.104, *t*(75) = 8.133, *p* < .001, [3.136, 3.548], *d* = 0.933; see Figure 3).

To test the hypothesis that children have weaker expectations of empathy for rivals than friends, we fit a linear regression model of children’s responses in the Empathy Block. Affiliation type (friends vs. rivals), outcome type (positive vs. negative), and the interaction between affiliation type and outcome type were included in the model as fixed effects. Affiliation type and outcome type were dummy-coded and mean-centered. We preregistered a mixed-effect model that included participant as a random effect, however this model returned a singular fit convergence issue due to the participant-level random effect variance being close to zero. So, we removed the random effect from the full Empathy Block model. The resulting full model was as follows:

response ∼ affiliation.type + outcome.type + affiliation.type * outcome.type

Using nested model comparisons, we then compared this full model to models that excluded each factor individually to test how much the excluded factor contributed to explaining the variance in children’s predictions of empathy. We found a main effect of affiliation type, *F*(1, 301) = 107.46, *p* < .001 – children were more likely to predict empathy for friends (*M* = 3.503, *SEM* = 0.070) than for rivals (*M* = 2.349, *SEM* = 0.098). We also found a main effect of outcome type, *F*(1, 301) = 13.338, *p* < .001 – children were more likely to predict empathy following negative outcomes (*M* = 3.132, *SEM* = 0.087) than for positive outcomes (*M* = 2.727, *SEM* = 0.103). There was also an interaction between affiliation type and outcome type, *F*(1, 301) = 42.883, *p* < .001. Using post-hoc paired-sample t-tests with Bonferroni adjustments (*α* = .0125, .05 divided by 4 tests), we found that children’s predictions of empathy following negative outcomes did not differ significantly for friends versus rivals (*M* = 2.920, *SEM* = 0.138), once corrected for multiple comparisons, *t*(73) = 2.156, *p* = .034, 95% CI [0.032, 0.806], *d* = 0.251, but that children predicted significantly more empathy from friends (*M* = 3.662, *SEM* = 0.092) than rivals (*M* = 1.792, *SEM* = 0.105) following positive outcomes, *t*(76) = 12.236, *p* < .001, [1.566, 2.175], *d* = 1.394. Within affiliation type, children’s predictions of empathy for a friend were not significantly different across positive and negative outcome types, once corrected for multiple comparisons, *t*(73) = −2.269, *p* = .026, [−0.593, −0.039], *d* = −0.260, but children predicted significantly more empathy for a rival who experienced a negative vs. positive outcome, *t*(74) = 6.770, *p* < .001, [0.790, 1.450], *d* = 0.781. Our data support the conclusion that children selectively predict empathic happiness for friends but not rivals following positive outcomes but expect empathic concern for both friends and rivals following negative outcomes.

**Figure 4.**
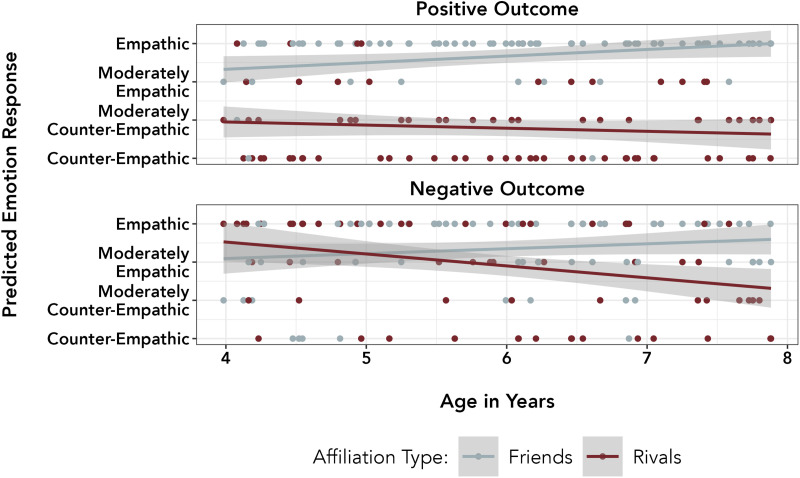
Children’s responses in the Empathy Block by age in years.

To investigate whether this pattern of reasoning changed across the age range in our sample, we conducted an exploratory analysis of all data from the Empathy Block, adding a continuous age variable and the associated two- and three-way interactions as fixed effects to the full model described above. A nested model comparison found a significant three-way interaction between age, affiliation type, and outcome type, *F*(4, 297) = 4.438, *p* = .002 (Figure 4). Post-hoc pairwise comparisons with Bonferroni adjustments (*α* = .0125, .05 divided by 4 tests) showed that the only significant difference in slopes was between predictions of empathy for friends and rivals following negative outcomes, estimate_diff_ = 0.440, *t*(297) = 3.389, *p* < .001, 95% CI [0.184, 0.695] – as children got older they were more likely to predict empathy for friends and counter-empathy for rivals after negative events. All other *t*s < 1.970 and *p*s > .050.

We also preregistered an exploratory analysis of only the older half of the sample, children 6 or 7 years of age, to assess if joint reasoning about vicarious emotions and social relationships is more robust in older children. Using the same set of nested linear regression comparisons as in the main analysis (i.e. without the continuous age factor), we again found a main effect of affiliation type on participants’ empathy predictions, *F*(1, 151) = 95.017, *p* < .001, and an interaction between affiliation type and outcome type such that empathy predictions varied more by affiliation type following positive vs. negative outcomes, *F*(1, 151) = 15.185, *p* < .001. However, post-hoc *t*-tests showed that in this sample subset, older children did predict more empathy following a negative outcome from a friend (*M* = 3.410, *SEM* = 0.120) than from a rival (*M* = 2.553, *SEM* = 0.202), *t*(37) = 3.141, *p* = .003, 95% CI [0.299, 1.385], *d* = 0.510. We followed up on this result by comparing older and younger children’s empathy predictions for each of the four story types and found that the only reliable difference was that older children predicted less empathy for rivals’ negative outcomes compared to younger children, *t*(73) = −2.82, *p* = .006, [−1.270, −0.219], *d* = 0.652. Younger children’s predictions of empathy for a rival’s negative outcome were greater than chance (2.5 on our scale), *M* = 3.297, *SEM* = 0.168, *t*(36) = 4.737, *p* < .001, [2.956, 3.639], *d* = 0.779, while older children’s predictions of empathy in the same situation were not significantly different from chance, *M* = 2.553, *SEM* = 0.202, *t*(37) = 0.260, *p* = .796, [2.143, 2.962], *d* = 0.042. Thus children’s expectation of empathic concern for rivals appears to decrease with development.

### Affiliation Block

To test if children use observations of empathic and counter-empathic responses to infer relationships, we fit the preregistered mixed-effect model of participants’ responses in the Affiliation Block. Emotion type (empathic response vs. counter-empathic response), outcome type (positive vs. negative), and the interaction between emotion type and outcome type were included in the model as fixed effects. Participant was included as a random effect. Emotion type and outcome type were dummy-coded and mean-centered. The full model was as follows:

response ∼ emotion.type + outcome.type + emotion.type * outcome.type + (1 | participant)

**Figure 5.**
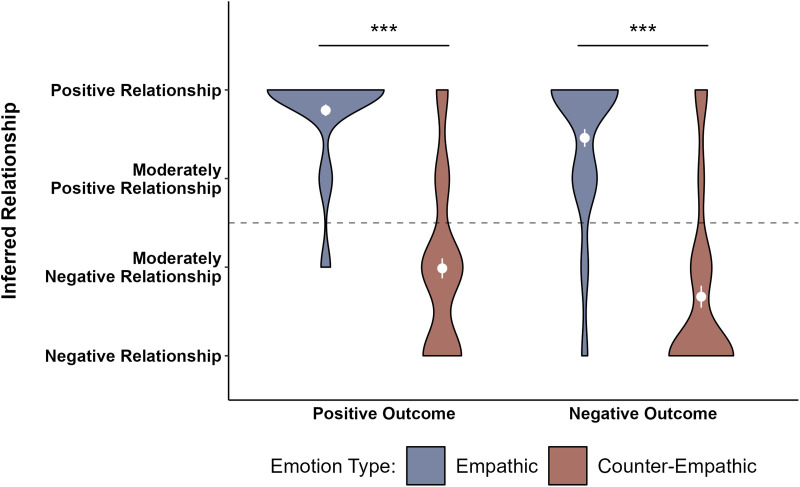
**Children’s responses for each question in the Affiliation Block.** The means and standard errors are plotted in white. The dashed line represents the mid-point of the scale (2.5). Significance asterisks come from nested model comparisons of mixed linear models testing for a main effect of emotion type. ***p < .001.

We used nested model comparison to compare the full model to models that excluded each fixed effect factor individually to test how much the excluded factor contributed to explaining the variance in children’s relationship inferences. We found a main effect of emotion type, *χ*^2^(1) = 219.09, *p* < .001 – children inferred a positive relationship more following an empathic response (*M* = 3.616, *SEM* = 0.061) than a counter-empathic response (*M* = 1.832, *SEM* = 0.084). We also found a main effect of outcome type, *χ*^2^(1) = 11.136, *p* = .001 – children were more likely to infer a positive relationship following positive outcomes (*M* = 2.878, *SEM* = 0.098) than for negative outcomes (*M* = 2.582, *SEM* = 0.109). There was no interaction between emotion type and outcome type, *χ*^2^(1) = 0.034, *p* = .853. In line with our predictions, children were more likely to infer a positive relationship between two characters when they observed one character respond empathically, rather than counter-empathically, to the other’s outcome, regardless of whether that outcome was a good or bad one (Figure 5).

**Figure 6.**
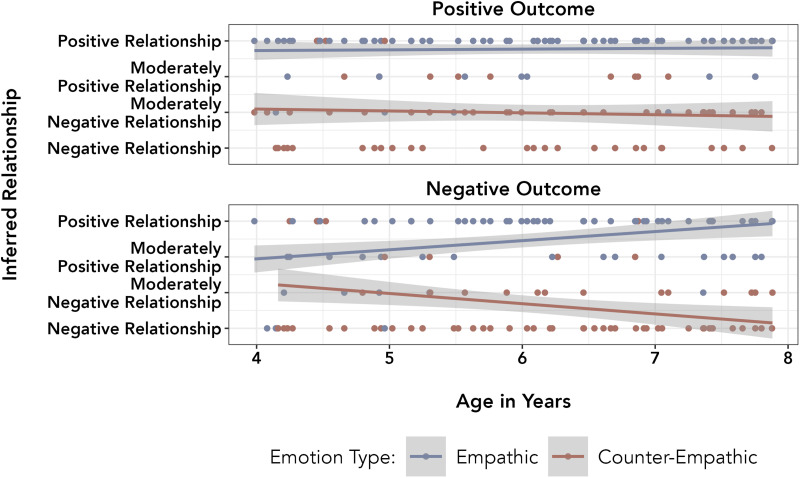
Children’s responses in the Affiliation Block by age in years.

As in the empathy block, we ran an exploratory analysis with a continuous age variable and related interactions as predictors for the Affiliation Block. There was a significant three-way interaction between empathic type, outcome type, and age, *χ*^2^(4) = 21.850, *p* < .001 (Figure 6). Post-hoc pairwise comparisons with Bonferroni adjustments (*α* = .0125, .05 divided by 4 tests) showed that the only significant difference in slopes was between relationship inferences from empathy and counter-empathy following negative outcomes, estimate_diff_ = 0.522, *t*(215) = 4.648, *p* < .001, 95% CI [0.301, 0.743]. As children got older they were more likely to infer positive relationships from empathy and negative relationships from counter-empathy after negative events. All other *t*s < 2.130 and *p*s > .034. By including the full set of age ranges, we found that children’s inferences about relationships from vicarious emotions following negative outcomes become stronger with age.

However, there are several reasons to see the developmental change in this case as incremental, rather than qualitative. First, the preregistered analysis of the older half of the age group revealed the same pattern of effects as in the full dataset: In the older children’s responses to the Affiliation Block, we again found a main effect of empathic type, *χ*^2^(1) = 190.51, *p* < .001 – children inferred a positive relationship more following an empathic response (*M* = 3.753, *SEM* = 0.059) than a counter-empathic response (*M* = 1.662, *SEM* = 0.097). We also found a main effect of outcome type, *χ*^2^(1) = 8.407, *p* = .004 – older children were still more likely to infer a positive relationship following positive outcomes (*M* = 2.859, *SEM* = 0.137) than for negative outcomes (*M* = 2.553, *SEM* = 0.149), as in the full dataset. There was no interaction between empathic type and outcome type, *χ*^2^(1) = 2.894, *p* = .089. Second, an analysis of only the younger half of the sample also found the same pattern: a main effect of empathic type, *χ*^2^(1) = 62.447, *p* < .001 – younger children inferred a positive relationship more following an empathic response (*M* = 3.464, *SEM* = 0.108) than a counter-empathic response (*M* = 2.030, *SEM* = 0.140); a main effect of outcome type, *χ*^2^(1) = 4.189, *p* = .041 – children were still more likely to infer a positive relationship following positive outcomes (*M* = 2.900, *SEM* = 0.141) than for negative outcomes (*M* = 2.615, *SEM* = 0.162), and no interaction between empathic type and outcome type, *χ*^2^(1) = 1.238, *p* = .266. In particular, 4- and 5-year-old children were more likely than chance to infer positive relationships from empathic responses to both positive outcomes, *t*(34) = 11.554, *p* < .001, 95% CI [3.501, 3.928], *d* = 1.953, and negative outcomes *t*(33) = 3.858, *p* < .001, [2.834, 3.578], *d* = 0.661, and more likely than chance to infer negative relationships from counter-empathic responses to both positive outcomes *t*(34) = −2.358, *p* = .023, [1.729, 32.443], *d* = −0.399, and negative outcomes *t*(30) = −2.367, *p* = .025, [1.509, 2.427], *d* = −0.425.

#### Exploratory Preregistered Order Analyses.

Across participants, outcome type order and affiliation type order (Empathy Block) or empathy type order (Affiliation Block) were counterbalanced. To examine any effect this may have had on children’s responses, we again used a nested model comparison to determine whether the addition of fixed order effects improved the model’s fit. There were no order effects observed in either the Empathy Block, *F*(2, 299) = 0.847, *p* = .430, or the Affiliation Block, *χ*^2^(2) = 0.991, *p* = .609. Additional preregistered exploratory analyses can be found in Supplemental Information.

## DISCUSSION

These data support the hypothesis that children can engage in systematic joint reasoning about social relationships and vicarious emotions (Smith-Flores & Powell, 2023). This includes both inferring social relationships from the information provided by empathic and counter-empathic responses (Affiliation Block) and making relationship-based predictions about vicarious emotions following positive outcomes (Empathy Block). The one context in which children did not display integrated reasoning about relationships and vicarious emotions across the full age range tested was when predicting one person’s response following someone else’s negative experience. In this context, children in the full sample expected others to express similar levels of empathy regardless of their relationships, though there was exploratory evidence that this varied between younger and older participant age groups. This failure may reflect a lack of understanding about the role negative relationships play in schadenfreude. However, given children’s predictions of counter-empathy following rivals’ good outcomes and their robust tendency to infer negative relationships from counter-empathy regardless of outcome valence, we suspect this result instead reflects young children’s general tendency to predict that people will behave normatively, rather than counter-normatively (DeJesus et al., 2014; Kalish & Shiverick, 2004; Powell & Smith, 2013; Shtulman & Phillips, 2018). Both adults and children consider most expressions of schadenfreude to be inappropriate, even toward disliked individuals (e.g., Geraci et al., 2021; Paulus et al., 2020; Wang & Todd, 2021). Thus our participants may have predicted an observer would respond empathically to a rival’s suffering because this is what they believe the observer *should* do. Children’s responses in the Affiliation Block also changed with age, becoming less likely to fall into the “moderate” categories of relationships when the emotions were a result of negative outcomes, but from the youngest ages children still used empathy and counter-empathy to make opposite relationship inferences. We conclude that, in addition to supporting our three primary hypotheses, the data are most consistent with the third possible developmental pattern laid out in the introduction: from the earliest ages tested, children already provided evidence of understanding the link between relationships and vicarious emotions, but the expression of this knowledge interacts with other aspects of cognitive development, including changes in modal reasoning.

Children’s ability to both predict vicarious emotion from relationships and infer relationships from vicarious emotion is consistent with the possession of an intuitive theory of psychology that connects relationship concepts to the causes of emotion. This proposal builds on work demonstrating that adults and children reason about emotions as the product of appraisals (Ong et al., 2019; Saxe & Houlihan, 2017; Wu et al., 2018), as well as the theory that early relationship concepts involve positing that relationship partners find the well-being of their affiliates rewarding (Hamlin et al., 2013; Powell, 2022). By connecting relationships to emotions through the reward that one person feels when good things happen to their friends (or bad things happen to their foes), or the sadness they feel in the opposite circumstances, this “adopted utility calculus” framework can support both forward and reverse inferences about how social relationships affect vicarious emotions (Smith-Flores & Powell, 2023). The unexpected finding that children inferred somewhat less positive relationships following negative versus positive outcomes is also consistent with probabilistic reasoning over an intuitive theory: Because children expect empathy to be normative and thus common following negative outcomes, (1) this empathy is potentially less convincing evidence of a close relationship than positive empathy and (2) counter-empathy for the negative outcome (i.e., schadenfreude) is more surprising and thus better evidence of a highly negative relationship than counter-empathy for the positive outcome. However, this is not the only possible basis for the pattern of reasoning observed here. Future research should investigate the integration of vicarious emotion reasoning with other components of intuitive psychology, including reasoning about ignorance or false belief, to test this account.

Relative to recent investigations of reasoning about vicarious emotions in preverbal infants, the current findings suggest both continuity and development. In a series of experiments, differences in 10- to 11-month-old infants’ looking time indicated that they were surprised if a friendly observer responded to an actor’s success with sadness, relative to a happy response. In contrast, when events depicted a rival observer watching the actor succeed, infants’ looking provided no evidence for an expectation of either a happy or a sad vicarious response (Smith-Flores et al., 2023). Thus, like the children in the current study, infants use social relationship information to inform their expectations about vicarious emotions after positive outcomes. However, infants did not use negative relationship information to predict counter-empathy from the observer following the actor’s success. In contrast, children in the current study, across all ages, predicted counter-empathy from rivals following positive events. Future research should more fully investigate the development of reasoning about counter-empathy. Infants’ lack of expectation may reflect a broader difficulty with reasoning about negative emotions, which extends to failures to predict negative emotional responses to direct experiences (Ruba et al., 2019, 2020; Skerry & Spelke, 2014). Alternatively, an understanding of empathic vicarious emotions may precede that of counter-empathic vicarious emotions, perhaps due to the difficulty of representing one person’s desire for the other to *not* achieve their goals (Feiman et al., 2015).

These findings provide insight into children’s reasoning about others’ social interactions and highlight children’s sensitivity to others’ displays of vicarious emotions. Children’s own empathic behaviors have been shown to play an important role in their ability to form positive social relationships (Brown & Fredrickson, 2021; Denham et al., 2003). Several theories regarding the development of emotional competence posit that empathy and emotion reasoning must develop together in order for children to achieve optimal social functioning (Hare et al., 2022; Hare & Parent, 2022; Taylor et al., 2017). Children with higher levels of emotion reasoning (i.e., the ability to correctly identify others’ emotions, also referred to as “cognitive empathy”; see Ruba & Pollak, 2020), have more positive social interactions and engage in more prosocial behaviors (Ensor et al., 2011). In clinical populations, training emotion recognition enhances empathic feelings and behavior (Teding van Berkhout & Malouff, 2016). Children’s developing understanding that vicarious emotions convey information about social relationships, displayed in the current findings, may also play a role in children’s own effective use of expressions of empathy. Our experiment finds that, at the group level, children readily predict empathy for friends’ successes and suffering, but it does not sensitively capture individual differences in children’s reasoning. Future research should explore connections to children’s own empathic tendencies and additional social–emotional competencies.

Finally, empathic and counter-empathic responses are rich sources of information about others’ prosociality, such as their propensity to help or provide comfort to those in distress (Batson, 2013; Bruneau et al., 2017; Eisenberg et al., 1993; Morelli et al., 2015). In this experiment, we tested whether children could infer relationships from observations of empathic responses. Children inferred that empathy signaled positive relationships rather than negative ones. In other work, children have been found to prefer and reward characters who comforted another character, suggesting that children may evaluate individuals who display empathic behaviors more favorably than non-empathic individuals (Geraci et al., 2021). However, it remains unknown whether children’s positive evaluations carry over into their selection of social partners for themselves. In one case, children may select empathizers if they need someone’s help, someone to play with, or someone to learn from when no other information is available. However, counter-empathy may also be a sign of a potential social partner if the counter-empathy is directed toward someone the child does not like. Recent work found that adults do prefer those who are kind and trustworthy over those who are not, but also that adults want friends who will be more positive to them than others and even be less prosocial to their enemies (Krems et al., 2023). Future work may explore how children evaluate empathizers and counter-empathizers as potential social partners and the nuances of such evaluations.

In sum, this experiment supports an early-developing social cognitive framework for joint reasoning about others’ relationships and vicarious emotions (Smith-Flores & Powell, 2023). This work gives rise to a number of questions about how children’s reasoning about other people carries over to their own social competencies and decision-making.

## AUTHOR CONTRIBUTIONS

Alexis Smith Flores: Conceptualization, Methodology, Formal analysis, Visualization, Writing—Original draft, Writing—Review & editing. Gabriel Bonamy: Investigation, Methodology, Writing—Original draft. Lindsey Powell: Conceptualization, Funding acquisition, Methodology, Formal analysis, Supervision, Writing—Review & editing.

## ACKNOWLEDGMENTS

We would like to thank the families who participated in these studies and Naomi Batarse, Ana Correa Avila Robb, and Marissa Garcia for assistance with data collection and coding.

## FUNDING STATEMENT

This work was supported by a grant from the Sanford Institute for Empathy and Compassion and start-up funding provided by UC San Diego to Lindsey Powell, and a Predoctoral Ford Fellowship awarded to Alexis Smith-Flores.

## DATA AVAILABILITY STATEMENT

This study was preregistered before data collection began. The research plan (including initial hypotheses and the analysis plan), materials and stimuli, data, and code used in the analysis are available on OSF: https://osf.io/d57ny.
