# Supplementary material for: Children’s Reasoning About Empathy and Social Relationships
Source: Open Mind (Camb). 2023 Oct 27;7:837–54. doi: 10.1162/opmi_a_00109 (PMC10631796; doi:10.1162/opmi_a_00109)
Supplement: Supplementary file 1 [file opmi-07-837-s001.docx]

Supplemental Information

**Repeated manipulation checks and exclusions**

Participants who said they would be equally happy to receive their favorite and least favorite snacks were excluded from the Empathy Block analysis, and participants who said they liked their favorite and least favorite characters the same amount were excluded from the Affiliation Block analysis. Table S1 reports the number of participants excluded from each block for these reasons.

|  | **Failed Warm-up Trial**  (number of children) | **4- & 5-year-olds** | **6- & 7-year-olds** |
| --- | --- | --- | --- |
| **Empathy** | 2 | 1 | 1 |
| **Affiliation** | 5 | 4 | 1 |

**Table S1.** Full block exclusion information.

***Individual story repeated checks and exclusions***

In the Empathy Block, to ensure children remembered the two characters’ affiliation, the researcher asked a manipulation check question following the story (e.g., “Remind me, are Ryan and Chris friends?”). Children who did not answer the manipulation check correctly were told the story again and had the manipulation check repeated. Children who did not answer the manipulation check correctly a second time were excluded from that question’s analysis (Table S2).

In the Affiliation Block, to ensure children remembered the emotion displayed by the emoter, the researcher then asked a manipulation check question (e.g., “Remind me, how does Brent feel?”). Children who did not answer the manipulation check correctly were told the story again and had the manipulation check repeated. Children who did not answer the manipulation check correctly a second time were excluded from that question’s analysis (Table S2).

**Exploratory Pregregistered Analyses**

***Skill Based Outcomes***

We preregistered an exploratory hypothesis about whether children considered outcomes differently when they were the result of a competitive scenario (e.g., running a race) or an event that occurred in the absence of competition (e.g., losing a favorite toy). However, as a result of our counterbalancing we did not fully cross event type (competition vs. not competition) with the different outcome and affiliation types, so we were unable to conduct a nested model comparison in the Empathy Block with event type as a fixed effect as initially preregistered.

| **Block** | **Affiliation/Emotion Information** | **Outcome for Target** | **Repeated Manipulation Check**  (number of children) | **Failed Manipulation Check**  (number of children) |
| --- | --- | --- | --- | --- |
| **Empathy** | Friends | Negative | 6 | 1 |
|  | Friends | Positive | 6 | 0 |
|  | Rivals | Negative | 1 | 1 |
|  | Rivals | Positive | 3 | 0 |
| **Affiliation** | Empathy | Negative | 1 | 1 |
|  | Empathy | Positive | 1 | 0 |
|  | Counter-Empathy | Negative | 7 | 5 |
|  | Counter-Empathy | Positive | 3 | 0 |

**Table S2.** Individual story repeat checks and exclusion information.

***Children’s flexible reasoning across blocks***

As an additional exploratory analysis we investigated the coherence of children’s reasoning by asking whether children answered similarly to the matched stories across blocks. For example, one story in the Affiliation Block featured a positive outcome and an empathic response and we hypothesized that children would infer that the characters have a positive relationship. In the Empathy Block a similar story exists where we hypothesized children would predict an empathic response from a character who witnessed their friend have a positive outcome. This type of similarity (based on the 2 x 2 of outcome valence and social valence) results in 4 matched story types.

We preregistered an exploratory phi-correlation to test this relationship in each of the 4 matched story types. We matched the stories and transformed children’s responses to binary outcomes (i.e., predictions of both highly and moderately empathic responses from the observer were coded as a 1, while predictions of both highly and moderately counter-empathic responses were coded as a 0; Figure S1). To test whether there was a relationship between children’s reasoning across the matched story types, we conducted a phi-correlation and an accompanying McNemar test on each of the matched four-story types. In the positive outcome & friends/empathic response matched story types there was no relationship between children’s responses across the blocks, ɸ = -0.08. The McNemar test determined that the proportion of children who inferred friendship from empathy did not differ across children who predicted empathy versus counter-empathy for a friend, **𝛘** ^2^(1) = 0.00, *p* = 1.00. In the negative outcome & friends/empathic response matched story type there was also no relationship between children’s responses across the blocks, ɸ = -0.12; there was again no difference in the proportion of children who inferred friendship given a prediction of empathy or counter-empathy in the other block, **𝛘** ^2^(1) = 0.00, *p* = 1.00. In each of these cases, the lack of a relationship in response patterns may reflect the fact that children were close to ceiling performance in each block: Fewer than 10% of children predicted counter-empathy for a friend, and fewer than 10% of children inferred rivalry following an empathic response. In the positive outcome & rivals/counter-empathic response matched story type there was also no relationship between children’s responses across the blocks, ɸ = 0.08; there was no difference in the proportion of children who inferred rivalry from counter-empathy across those who predicted empathy vs. counter-empathy, **𝛘** ^2^(1) = 0.042, *p* = .838. Finally, in the negative outcome & rivals/counter-empathic response matched story type there was a weak positive relationship in children’s responses across the blocks, ɸ = 0.21; there was a significant difference in the proportion of children who inferred rivalry from counter-empathy when comparing children who predicted counter-empathy and those who predicted empathy for a rival’s misfortune, **𝛘** ^2^(1) = 22.781, *p* < .001. Children who predicted counter-empathy almost always inferred rivalry from observed counter-empathy (92%), while children who predicted empathy were somewhat less likely to do so (75%).

| 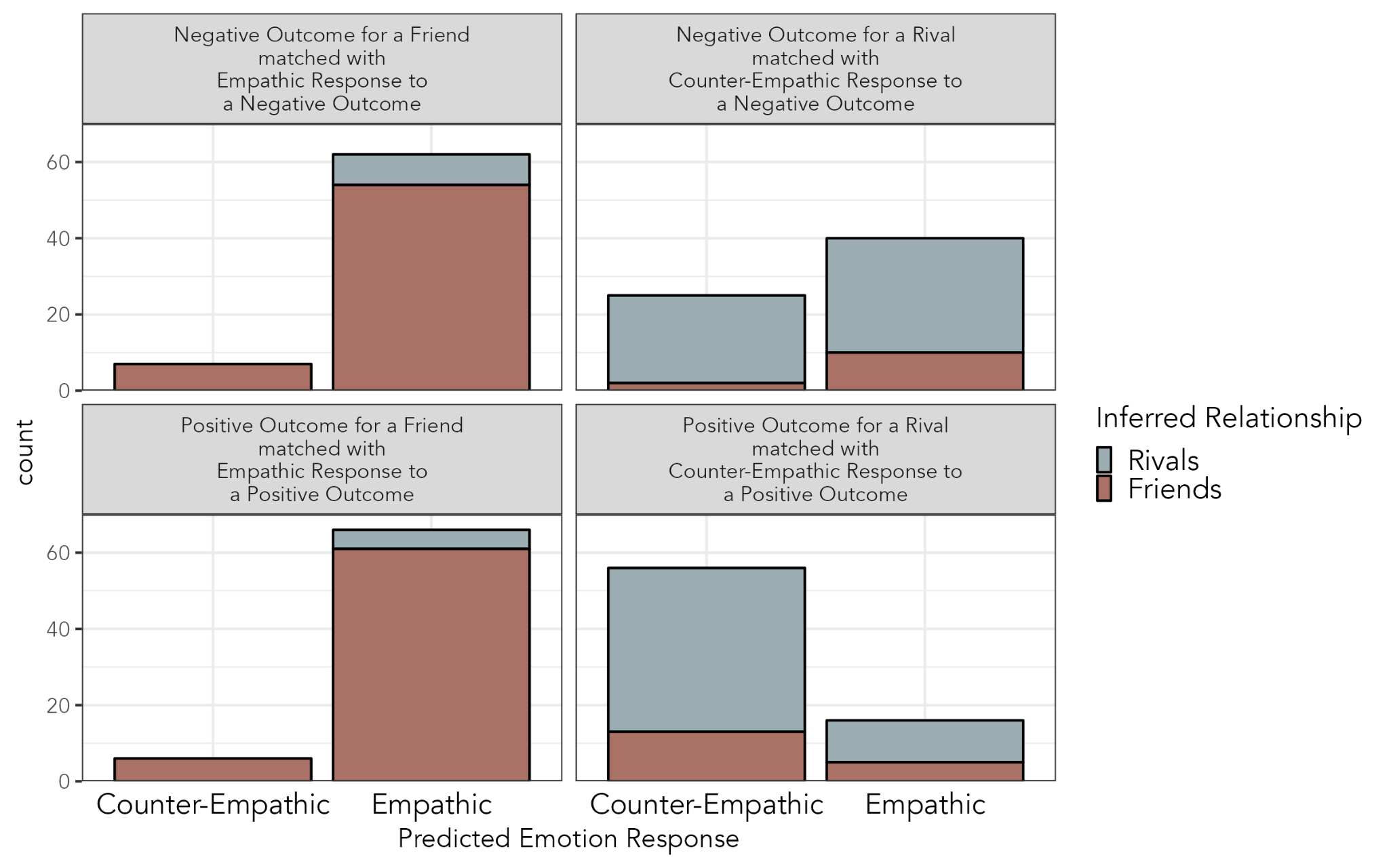 |
| --- |
| **Figure S1.** Children’s binary responses on matched story types across blocks. |
